# Heat-Induced Cytokinin Transportation and Degradation Are Associated with Reduced Panicle Cytokinin Expression and Fewer Spikelets per Panicle in Rice

**DOI:** 10.3389/fpls.2017.00371

**Published:** 2017-03-17

**Authors:** Chao Wu, Kehui Cui, Wencheng Wang, Qian Li, Shah Fahad, Qiuqian Hu, Jianliang Huang, Lixiao Nie, Pravat K. Mohapatra, Shaobing Peng

**Affiliations:** ^1^National Key Laboratory of Crop Genetic Improvement, MOA Key Laboratory of Crop Ecophysiology and Farming System in the Middle Reaches of the Yangtze River, Huazhong Agricultural UniversityWuhan, China; ^2^Hubei Collaborative Innovation for Grain Industry, JingzhouChina; ^3^School of Life Sciences, Sambalpur UniversitySambalpur, India

**Keywords:** cytokinin oxidase/dehydrogenase, cytokinin synthetic enzymes, heat stress, panicle cytokinins, panicle size, rice (*Oryza sativa*), root xylem sap

## Abstract

Cytokinins (CTKs) regulate panicle size and mediate heat tolerance in crops. To investigate the effect of high temperature on panicle CTK expression and the role of such expression in panicle differentiation in rice, four rice varieties (Nagina22, N22; Huanghuazhan, HHZ; Liangyoupeijiu, LYPJ; and Shanyou63, SY63) were grown under normal conditions and subjected to three high temperature treatments and one control treatment in temperature-controlled greenhouses for 15 days during the early reproductive stage. The high temperature treatments significantly reduced panicle CTK abundance in heat-susceptible LYPJ, HHZ, and N22 varieties, which showed fewer spikelets per panicle in comparison with control plants. Exogenous 6-benzylaminopurine application mitigated the effect of heat injury on the number of spikelets per panicle. The high temperature treatments significantly decreased the xylem sap flow rate and CTK transportation rate, but enhanced cytokinin oxidase/dehydrogenase (CKX) activity in heat-susceptible varieties. In comparison with the heat-susceptible varieties, heat-tolerant variety SY63 showed less reduction in panicle CTK abundance, an enhanced xylem sap flow rate, an improved CTK transport rate, and stable CKX activity under the high temperature treatments. Enzymes involved in CTK synthesis (isopentenyltransferase, LONELY GUY, and cytochrome P450 monooxygenase) were inhibited by the high temperature treatments. Heat-induced changes in CTK transportation from root to shoot through xylem sap flow and panicle CTK degradation via CKX were closely associated with the effects of heat on panicle CTK abundance and panicle size. Heat-tolerant variety SY63 showed stable panicle size under the high temperature treatments because of enhanced transport of root-derived CTKs and stable panicle CKX activity. Our results provide insight into rice heat tolerance that will facilitate the development of rice varieties with tolerance to high temperature.

## Introduction

The global mean surface temperature increased rapidly and considerably during the 20th century, and a further increase of 0.3–4.8°C is predicted by the end of the 21st century (Pachauri et al., 2014). Notably, nighttime temperature has increased more rapidly than has daytime temperature (Peng et al., 2004; Elagib, 2010). High temperature extremes and warmer nights are likely to become more frequent and intense in the near future (Mika, 2013; Wang et al., 2014).

Rice plants are highly susceptible to high temperature stress, especially during the reproductive stage (Moldenhauer et al., 2001; Jagadish et al., 2015), during which high temperature may severely reduce grain yield. An increase of 1–4°C reduced rice grain yield by 0–49%; grain yield decreased 14% for every 1°C increase in temperature (Singh et al., 2009). Another study showed that an increase of 1°C in nighttime temperature reduced rice grain yield by 10% (Peng et al., 2004). Heat-induced yield reduction is largely attributed to adverse effects on yield components (Jagadish et al., 2015).

During the early reproductive phase of rice, which includes the processes of panicle initiation and development, high temperature events, including relatively warm nights, reduced the number of spikelets (Wei et al., 2010; Wu et al., 2016). During the middle and late reproductive phases, during which heading and grain filling occur, high temperature events reduce the grain filling rate and grain weight in rice (Jagadish et al., 2015). Several studies have assessed the agronomic, physiological, and molecular aspects of the negative impact of heat stress on rice production, especially at the flowering and grain filling stages (Shi et al., 2013; Jagadish et al., 2015). However, understanding of the physiological aspects of the impact of high temperature on panicle differentiation and spikelet formation during the early reproductive phase is limited (Wang et al., 2015).

In higher plants, cytokinins (CTKs) regulate numerous biological processes, including shoot growth and development, differentiation, and responses to environmental stresses (Ha et al., 2012; Zwack and Rashotte, 2015). In rice, CTKs also regulate the number of spikelets (Kyozuka, 2007). Rice varieties with small panicles had correspondingly low panicle CTK abundance, but exogenous CTK application to plants gradually increased the number of spikelets (Ashikari et al., 2005; Ding et al., 2014). These results show that CTKs play crucial roles in panicle formation and spikelet differentiation in rice.

Cytokinin accumulation in the aerial organs of plants is determined by the rate of importation of root-synthesized CTKs, local CTK synthesis, and local CTK catabolism (Kudo et al., 2010). Root-derived CTKs are the main source of CTKs for aerial organs (Aloni et al., 2005), in which most CTKs are derived from the roots and transported to shoot via xylem sap (Yong et al., 2014). In *Betula pubescens*, CTK abundance in xylem sap was positively correlated with the growth rate of shoot and number of branches (Rinne and Saarelainen, 1994). CTK synthesis involves several enzymes, but isopentenylation of adenosine phosphate by isopentenyltransferases (IPT) to produce N^6^-(Δ^2^-isopentenyl) adenosine (iP) phosphate is the key step in CTK biosynthesis (Miyawaki et al., 2006). *Trans*-hydroxylation of the side-chain of iP-riboside to form *trans*-zeatin (tZ) riboside is catalyzed by cytochrome P450 mono-oxygenase CYP735A (Kiba et al., 2013). Additionally, LONELY GUY (LOG) proteins with phosphoribohydrolase activity are involved in the conversion of riboside 5′-monophosphate CTKs with low activity to high-activity forms such as iP (Kurakawa et al., 2007; Tokunaga et al., 2012). Degradation of CTKs is primarily catalyzed by cytokinin oxidase/dehydrogenase (CKX) (Kudo et al., 2010).

Suppressed CTK degradation and enhanced CTK biosynthesis contribute to local CTK accumulation in plant organs (Kudo et al., 2010). CTK metabolism influences panicle size in rice; relatively low CKX activity was associated with reduced panicle CTK degradation and a greater number of spikelets per panicle (Ashikari et al., 2005). And, others have reported that enhanced CTK biosynthesis is associated with large panicle size in rice. For example, expression levels of *IPT* and *LOG*, which are involved in local CTK synthesis in shoot meristem, were directly associated with the number of panicle spikelets (Kurakawa et al., 2007; Ding et al., 2014). In *Arabidopsis*, shoot development and growth were promoted by overexpression of *CYP735A* and retarded in loss-of-function *CYP735A* mutants (Kiba et al., 2013). However, few studies have simultaneously evaluated enzymes involved in CTK synthesis and catabolism, especially in rice, and it is not yet clear which processes and enzymes involved in CTK metabolism influence panicle CTK accumulation and panicle differentiation.

Cytokinins mediate plant responses to abiotic stresses, including heat stress (Ha et al., 2012). In rice, *Arabidopsis* and passion fruit, reduced CTK abundance in shoots in response to heat caused floret abortion, whereas exogenous CTK application to shoots mitigated the effects of heat injury on branches and florets (Sobol et al., 2014; Wu et al., 2016). CTK transportation via xylem sap is involved in heat stress tolerance (Ha et al., 2012; Zwack and Rashotte, 2015). In creeping bentgrass, application of zeatin riboside (ZR) to the root zone increased the abundance of shoot CTKs and alleviated heat stress injury following exposure to high soil temperature and high air temperature (Liu et al., 2002). However, Udompraset et al. (1995) found that root tZR was not involved in the development of heat tolerance in *Phaseolus vulgaris*. These findings show that the relationship between CTK transportation from root to shoot and heat tolerance is not well understood.

Changes in CTK metabolism are thought to be involved in adaptation by plants to various environmental stresses. Enzymes involved in CTK synthesis play roles in stress responses in several plant species. IPT, the key enzyme in the process of CTK synthesis, is involved in adaption to heat, drought, osmotic stress, and salt stress in maize, peanut, and *Arabidopsis* (Vyroubalová et al., 2009; Qin et al., 2011; Skalák et al., 2016). In rice, LOG expression is altered by various abiotic stress conditions, including heat stress, cold stress, drought, and salinity (Tripathi et al., 2012). CYP735A expression in rice was altered by exposure to cold and dehydration (Maruyama et al., 2014). Regulation of CTK abundance by CKX occurs in response to heat, cold, drought, and salinity in rice, maize, tobacco, and pea, respectively (Vaseva-Gemisheva et al., 2004; Vaseva et al., 2009; Vyroubalová et al., 2009; Tripathi et al., 2012; Lubovská et al., 2014). Accumulation of CTKs as a result of impaired degradation, enhanced local biosynthesis, and/or enhanced CTK transportation from roots, is favorable for adaption to abiotic stress (Ha et al., 2012). Therefore, assessing the effects of heat on CTK transportation from root to shoot via xylem sap and the activity levels of enzymes involves in CTK metabolism, such as IPT, LOG, CYP735A, and CKX, should illuminate the mechanisms underlying phytohormonal regulation of heat tolerance in rice.

Rice varieties display wide genotypic variation in the physiological response to heat stress (Tao et al., 2009; Shi et al., 2013). In this study, we investigated genotypic variation in physiological processes associated with CTK homeostasis in rice plants exposed to high temperature, as well as the relationship between these processes and spikelet number, with the goal of revealing the mechanisms underlying hormonal regulation of panicle size under heat stress during the early reproductive phase.

## Materials and Methods

### Crop Husbandry

Pot experiments were conducted during the rice growth season of 2013 at Huazhong Agricultural University, Wuhan, China (30°29′ N, 114°22′ E). Four varieties were used in this study: N22, HHZ, LYPJ, and SY63. The methods for crop husbandry, temperature treatments, and phytohormone determination were described as previous study (Wu et al., 2016).

After breaking dormancy at 50°C for 5 days, seeds were sown in plastic seeding trays with loam soil. Four three-leaf seedlings were transplanted into a 14 L plastic pot (28.5 cm height × 30 cm top diameter × 25 cm bottom circumference) containing a mixture of 17 kg soil (loam:sand, 2:1) and 12.5 g compound fertilizer (N:P_2_O_5_:K_2_O, 16%:16%:16%). Seedlings were thinned to three plants per pot 8 days after transplanting, and the main tillers were tagged. A total of 1.0 g urea was topdressed per pot 10 days after transplanting. The plants were flooded with water staying approximately 2 cm above soil surface from sowing to maturity. Each pot was manually rotated by 90° clockwise every 7 days to avoid positional effects. Pests, diseases, birds, and weeds were intensively controlled.

### High Temperature Treatments

The rice plants were randomly arranged with four replications. All plants were carefully cultivated under natural ambient conditions, after which they were moved to four temperature-controlled greenhouses at the start of panicle initiation (panicle emergence was observed visually). The greenhouses were equipped with a wetting machine, an air conditioner, two ventilators, and two sensors for monitoring relative humidity (RH) and air temperature.

**FIGURE 1:**
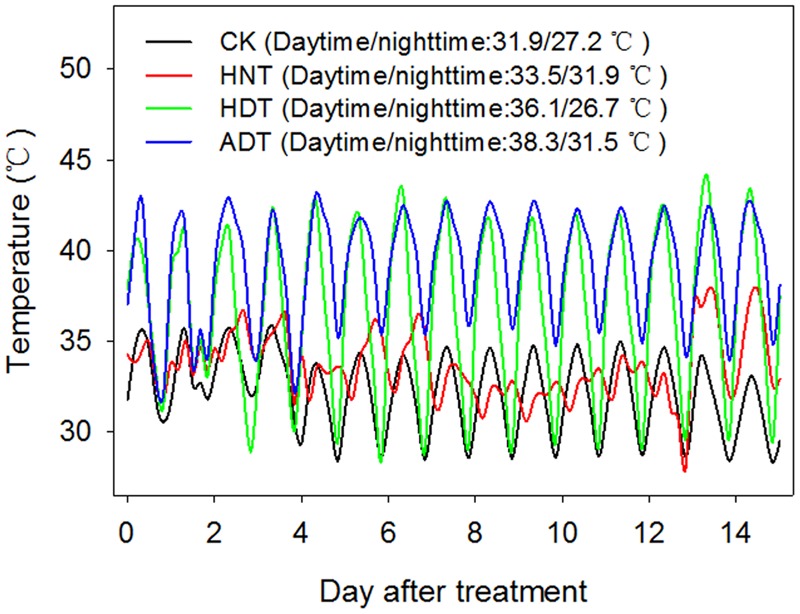
**Dynamic temperature records for the three high-temperature treatments and one control treatment**.

The four temperature treatments included a high nighttime temperature treatment (HNT) that imposed high temperature from 19.00 to 07.00 h, high daytime temperature treatment (HDT) that imposed high temperature from 07.00 to 19.00 h, high daytime plus nighttime temperature treatment (ADT) that imposed high temperature during the entire day, and control (CK) treatment that imposed a favorable temperature for rice plant growth during the entire day. RH was set at 80%. The air temperature and RH in the greenhouse were controlled by a central auto-controller (Auto-Greenhouse Monitoring and Data Management System, Version 3.00, Auto, China). Air temperature and RH% were recorded 5 cm above the rice canopy using a standalone sensor (HOBO, H08-003-02, Onset Computer Corporation, Bourne, MA, USA). The mean nighttime and daytime temperatures were 27.2 and 31.9°C under the CK treatment. The mean daytime temperature was 36.1°C under the HDT treatment (4.2°C higher than that of the CK treatment). The mean nighttime temperature was 31.9°C under HNT treatment (4.7°C higher than that of the CK treatment). The mean nighttime and daytime temperatures under the ADT treatment were 31.5 and 38.3°C, respectively (4.3 and 6.4°C higher than those of the CK treatment, respectively) (**Figure 1**).

The plants were exposed to the high temperature treatments for 15 days, after which the plants were removed and grown continuously under normal conditions until maturity, at which point panicle length was approximately 1.5–2.0 cm.

### Application of Exogenous Benzylaminopurine (BAP)

The solution of BAP (60 mg L^-1^), a synthetic CTK, was prepared by dissolving 60 mg BAP (Sigma-Aldrich, USA) in 1 mL of 1% (w/v) NaOH solution, following by dilution to a final volume of 1 L with double-distilled H_2_O. Two drops of 0.01% (v/v) Tween 20 was added as a surfactant, after which the solution was mixed thoroughly. The BAP solution was sprayed onto the stems of LYPJ (heat-sensitive) and SY63 (heat-tolerant) varieties under the ADT treatment (20 mL per plant per application). The BAP solution was applied twice: 1 day before the high temperature treatments and on the second day after the high temperature treatments.

### Determination of Yield Traits

To determine the number of spikelets per panicle and grain yield, three panicles of three main tillers were harvested at maturity from three plants grown in three pots for each replication. All spikelets were threshed from the panicles manually, and then collected to determine grain yield and yield components. Grain yield and other yield components were also calculated. The number of spikelets per panicle was referred to as the number of spikelets in the main tiller.

### Determination of CTKs in the Panicles, Roots, and Xylem Sap

For each replication, three young panicles of the main tillers were collected from three plants grown in three pots at the last day of exposure to the high temperature treatments. The panicles were frozen in liquid nitrogen and stored at -80°C. Next, xylem sap was collected from the same three plants during the night (19.00–07.00 h) as described by Arai-Sanoh et al. (2010). The number of tillers per plant was counted, after which the plants were cut approximately 8 cm above the soil level. Dead leaf sheathes in stubbles were cleared artificially, and the first droplet of exudation was wiped to avoid contamination. Polyethylene bags containing cotton wool (6–7 g) were attached to the cut ends and fixed with rubber bands, and collected root xylem sap for 12 h. The difference in weight of the cotton wool was considered as the weight of the collected root exudates. The root xylem sap flow rate (mg tiller^-1^ h^-1^) was calculated as the amount of collected sap per tiller divided by 12. Finally, roots were sampled and washed to allow collection of fresh white roots. The collected roots and exudates were stored at -80°C and used for measurements of CTK abundance.

### Extraction and Determination of CTKs

Cytokinins in the roots, xylem sap, and panicles were extracted and purified according to methods reported by Xie and Zhang (2001) and Hoyerová et al. (2006) and quantified according to the methods of Chou et al. (2000) using high performance liquid chromatography (HPLC) with some minor modifications.

For CTKs in the panicles and roots, frozen samples were cut into pieces and mixed completely. Next, 1 g of tissue was ground with 8 mL of cold extraction buffer (methanol:double-distilled H_2_O:formic acid, 15:4:1). The homogenates were transferred to 10-mL centrifuge tubes, incubated at 4°C for 12 h, and centrifuged at 12,000 × *g* for 20 min at 4°C, after which supernatants were collected. The pellets were subjected to phytohormone extraction twice as described above (e.g., 8 mL cold extraction buffer and centrifugation). All supernatants were pooled and condensed to 2 mL using a freeze-dryer (ALPHA 1-4 LD plus, Marin Christ, Osterode, Germany), after which 2 mL of petroleum ether was added to extract pigments and phenolics. The extraction was repeated three times. After removing the petroleum ether containing pigments and phenolics, the lower aqueous phase was freeze dried, and 3 mL of sodium acetate (1 mol/L, pH 8.0) was used to resuspend the samples as crude extracts for determination of various CTKs.

For CTK extraction in root xylem sap, 10 mL of xylem sap was collected in a centrifuge tube by extruding the cotton wool in which the xylem sap was collected. The xylem sap was centrifuged at 12,000 × *g* for 20 min at 4°C, after which 8 mL of the supernatant was collected and freeze-dried. Next, the sample was resuspended in 3 mL of sodium acetate (1 mol/L, pH 8.0). These solutions were designated as the crude extracts for determination of various CTKs.

For further CTK extraction, the crude extract (3 mL) was extracted with 1-butanol (3 mL) three times, after which the upper organic phase (1-butanol) containing CTKs was pooled together for CTK measurement. The lower aqueous phase was pooled, adjusted to pH 3.0, and extracted three times with ethyl acetate. The upper organic phase (ethyl acetate) was pooled as the mixture of IAA, GAs, and ABA.

All collected upper organic phases were pooled together for determining CTKs, IAA, GAs, and ABA. After freeze-drying, the residues were dissolved in 5 mL of methyl alcohol and purified using a C_18_-SepPak cartridge (Waters Corporation, Milford, MA, USA). The purified samples were freeze-dried and dissolved in 0.8 mL of methyl alcohol for phytohormone determination. The elution procedure, flow rate, column temperature, and detection wavelength for HPLC analysis were optimized. Based the optimized conditions, abundance of CTKs was measured using HPLC equipped with a C_18_ column (WondaCract ODS-2 C_18_ column; 4.6 mm × 250 mm, 5 μm) by a multi-step linear gradient elution (45 min) at a flow rate of 1.6 mL min^-1^. The column temperature was maintained at 45°C. The UV detection wavelength was 269 nm. The solutions for eluting according to the methods of Chou et al. (2000) with modification. The solutions for eluting include methanol (A), double-distilled H_2_O (B), and 4.5% acetic acid solution (C). The following protocol was used for gradient elution: 0 min, 0% A and 100% B; 17 min, 30% A and 70% B; 18 min, 40% A and 60% B; 22 min, 40% A and 60% B; 24 min, 35% A and 65% B; 25min, 35% A and 65% C; 35min, 100% B; 45min, 100% B.

The calibration standards were mixed in a CTK standard solution containing isopentenyladenine riboside-5’-monophosphate (iPMP), iP, N^6^-(Δ^2^-isopentenyl) adenosine riboside (iPA), tZ, and tZR (OlChemIm Ltd., Czechia). The calibration standards were prepared at concentrations of 5.7, 8.5, 11.4, 45.6, 91.1, and 182.3 ng mL^-1^ for each hormone standard in the mixed standard solution of five compounds. Calibration standard curves were repeated four times, after which a standard curve was calculated for each compound.

To evaluate recovery of extracted CTKs, a 1-g sample of panicle tissue was ground with 150 ng of each CTK standard mentioned above. The percentage recovery of tZ, tZR, iPMP, iP, and iPA was 109.4 ± 5.4, 97.8 ± 4.4, 62.1 ± 2.2, 96.7 ± 4.1, 97.2 ± 5.0%, respectively.

The concentration of each CTK in the roots and panicles was expressed as ng g^-1^ based on fresh weight, whereas the concentration in xylem sap was expressed as pg mL^-1^. The transport rate of each CTK via xylem sap (pg tiller^-1^ hr^-1^) was calculated by multiplying the xylem sap flow rate by the concentration of the corresponding CTK in xylem sap.

### Enzymatic Analysis of CKX, IPT, CYP735A, and LOG

#### Enzyme Extraction and Soluble Protein Determination

According to the methods described by Zalewski et al. (2010), a 0.5-g sample of young panicles was cut into pieces, powdered with liquid nitrogen using a hand mortar, and extracted with 6 mL of TRIS-HCl buffer (0.2 M, pH 8.0, containing 1 mM phenylmethylsulfonyl fluoride and 0.3% Triton X-100).

All debris was removed by centrifugation at 12,000 × *g* for 15 min at 4°C. The supernatants were collected and used for determinations of enzyme activity. The soluble protein concentration was evaluated using the method of Bradford (1976), with bovine serum albumin as the standard.

#### CKX Activity

The assay of CKX activity based on iP degradation was performed according to the methods of Frébort et al. (2002) with minor changes. A reaction mixture containing 0.2 mL of the enzyme extract, 0.2 mL iP (0.15 mM), 0.5 mL 2,6-dichlorophentolindophenol (0.5 mM), and 0.2 mL Tris/HCl buffer (75 mM, pH 8.5) was incubated at 37°C for 60 min, after which the reaction was stopped by the addition of 0.3 mL trichloroacetic acid (40%). The mixture was centrifuged at 18,000 × *g* for 30 min. HPLC analysis was used to quantify iP by measuring absorbance at 269 nm as described previously. CKX activity (nmol mg^-1^ protein h^-1^) was defined as the amount of iP (nmol) degraded by 1 mg protein per hour under the selected reaction conditions.

#### IPT Activity

The assay of IPT activity was performed according to the method of Takei et al. (2001a) with minor changes. The enzyme extract (0.2 mL) was incubated in 0.2 mL of the reaction mixture (1 M betaine, 20 mM triethanolamine, 50 mM KCl, 10 mM MgCl_2_, 1 mM dithiothreitol, 1 mg/mL bovine serum albumin, pH 8.0) with 0.2 mL adenosine monophosphate (1 mM) and 0.3 mL dimethylallylpyrophosphate (340 μM) at 25°C for 2 h, after which the reaction was stopped by the addition of 0.2 mL acetate (10%). When optimizing the conditions for the IPT activity determination, we had set six incubation times, i.e., 20, 30, 50, 60, 90, and 120 min, respectively, and found that the activity of IPT was highest when incubating for 2 h, and the activity did not detected when incubating for 20, 30, 50, 60. The mixture was centrifuged at 18,000 × *g* for 20 min. The supernatant was subjected to HPLC, and iPMP was quantified by measuring absorbance at 269 nm as described previously. The activity of IPT (nmol mg^-1^ protein h^-1^) was defined as the amount of produced iPMP (nmol) per 1 mg protein per hour under the selected reaction conditions.

#### LOG Activity

The assay of LOG activity was performed according to the method of Kurakawa et al. (2007) with minor changes. The enzyme extract (0.2 mL) was incubated in 0.2 mL of the reaction mixture (50 mM Tris-HCl, 1 mM MgCl_2_, 1 mM dithiothreitol, pH 6.5) with 0.08 mL iPMP (10 mM) at 30°C for 2 h. The reaction was terminated using 0.3 mL of cold acetone. The mixture was stored at -80°C for 30 min and centrifuged at 18,000 × *g* for 20 min. The supernatant was subjected to HPLC. The content of synthetic iP was quantified by measuring absorbance at 269 nm as described previously. LOG activity (nmol mg^-1^ protein h^-1^) was defined as the amount of iP produced per hour per mg protein under the selected reaction conditions.

#### CYP735A Activity

The assay of CYP735A activity was performed according to the method of Sasaki et al. (2013) with minor changes. The enzyme extract (0.2 mL) was incubated with 0.2 mL of the reaction mixture (100 mM sodium phosphate, 10% sucrose, 3 mM triphosphopyridine nucleotide, 1 mg/mL bovine serum albumin, pH 7.5) and 0.08 mL iPMP (10 mM) at 20°C for 2 h. The reaction was terminated by the addition of 0.2 mL of termination buffer (50 mM CHES-NaOH, 0.5 mM MgCl_2_, pH 10.0). The mixture was incubated with 0.01 mL of calf-intestine alkaline phosphatase (1 u/μL, Sigma) at 37°C for 40 min and centrifuged at 18,000 × *g* for 20 min. The supernatant was subjected to HPLC. The content of tZR was quantified by measuring absorbance at 269 nm and comparison with the standard curve for tZR. CYP735A activity (nmol mg^-1^ protein h^-1^) was defined as the amount of tZR produced per hour per mg protein under the selected reaction conditions.

### Statistical Analysis

In this study, tZ-type and iP-type CTKs were referred to as biologically active CTKs (aCTKs), including tZ, tZR, iPMP, iP, and iPA. Relative values for traits are presented in this study as a means of evaluating the responses of rice plants to high temperature treatments. A relative value was defined as the ratio of the value under a high temperature treatment to that under the control treatment for the same trait in the same variety. A relative value less or more than 1.0 indicated a decrease or increase in a trait under a high temperature treatment compared with that under the control condition, respectively. The absolute values of CTK contents, xylem sap flow rate, enzyme activities are presented as Supplementary Tables S1–S3, respectively. The absolute concentrations of CTKs in young panicle have already presented in our previous paper (Wu et al., 2016), in which we focused the relationship panicle differentiation and panicle CTK contents under high temperature treatments.

The mean of the relative value across the four replicates was used for analysis of variance and determination of significant differences by the least significant difference (LSD) test at *P* < 0.05 using Statistix 8.0 (Analytical Software, Tallahassee, FL, USA). Regression analysis was used to estimate the relationship among the investigated traits across four varieties and three high temperature treatments (*n* = 12) using Sigmaplot software (version 12.5; SPSS Inc., Chicago, IL, USA).

## Results

### Number of Spikelets per Panicle under High Temperature Treatments

**FIGURE 2:**
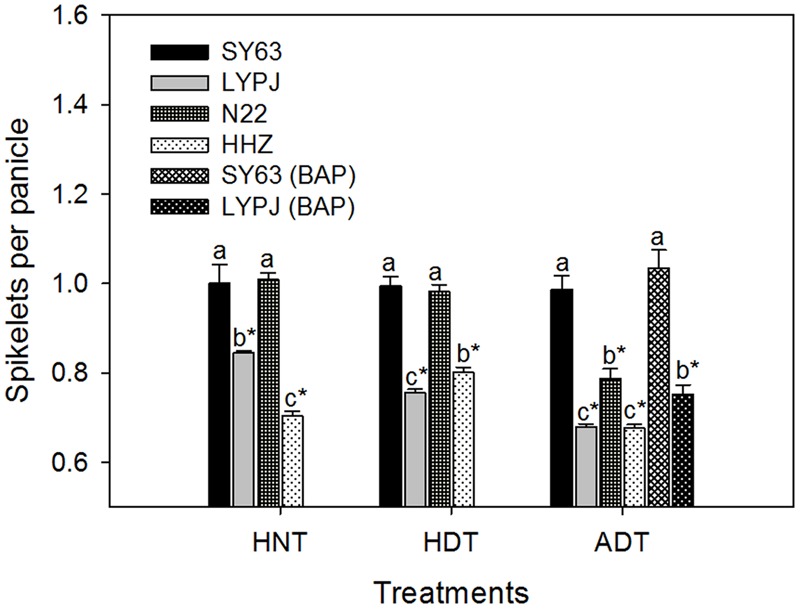
**Relative number of spikelets per panicle of rice varieties under high temperature treatments.** Data are presented as mean ± SD (*n* = 4). Different letters indicate significant differences among varieties under the same temperature treatment by a least significant difference (LSD) test at *P* < 0.05. Asterisks indicate significant differences when comparing the absolute mean of a trait under a given high temperature treatment with that under the control by LSD test at *P* < 0.05.

The absolute numbers of spikelets per panicle in N22, HHZ, LYPJ, and SY63 were 103, 203, 196, and 127 under control temperature treatment, respectively. As shown in **Figure 2**, the three high temperature treatments significantly reduced the number of spikelets per panicle in the HHZ and LYPJ varieties, but the largest reductions in the number of spikelets per panicle were observed under the ADT treatment. The number of spikelets per panicle was reduced by 19.9–32.3% in the HHZ variety and 15.6–32.0% in the LYPJ variety under the three high temperature treatments. For the N22 variety, the number of spikelets per panicle was reduced significantly only under the ADT treatment. For the SY63 variety, the number of spikelets per panicle was not affected significantly by the three high temperature treatments. The SY63 variety had more spikelets per panicle in comparison with the LYP9 and HHZ varieties under the three high temperature treatments, as well as more spikelets per panicle in comparison with the N22 variety under the ADT treatment.

Application of exogenous BAP increased the number of spikelets per panicle by 11% in the LYPJ variety and by 5% in the SY63 variety under the ADT treatment, in comparison with the same varieties under the ADT treatment without BAP application (**Figure 2**).

### Panicle CTK Content under High Temperature Treatments

In rice panicles, absolute concentrations of tZ+tZR, iPMP+iP+iPA, and aCTKs were 114, 223, and 336 ng/g in N22, 169, 208, and 376 ng/g in HHZ, 184, 257, and 441 ng/g in LYPJ, and 114, 238, and 353 ng/g in SY63 under control temperature treatment, respectively (Supplementary Table S1).

The HNT treatment significantly decreased the abundance of tZ+tZR, iPMP+iP+iPA, and aCTKs, with the exception of tZ+tZR in the N22 and SY63 varieties (**Table 1**). Similarly, the HDT and ADT treatments significantly reduced the abundance of the tested CTKs, with the exception of tZ+tZR in the SY63 variety. Generally, the largest reductions were found under the ADT treatment; on average across the four varieties, iP-type CTKs, tZ-type CTKs, and aCTKs in the panicles were reduced in abundance under ADT treatment by 57% (17–73%), 25.3% (0–39%), and 45.8% (16–57%), respectively. In general, the abundance of iP-type CTKs was reduced more than was the abundance of tZ-type CTKs. On average, iP-type CTKs and tZ-type CTKs in the panicles were reduced in abundance by 40.4% (15–73%) and 17.0% (0–39%), respectively, across the four varieties and three high temperature treatments (**Table 1**).

**Table 1:** Relative cytokinin (CTK) concentrations in panicles and roots under high temperature treatments.

Treatment	Variety	CTKs in the panicles			CTKs in the roots		
		tZ+tZR	iPMP+iP+iPA	aCTKs	tZ+tZR	iPMP+iP+iPA	aCTKs
HNT	N22	0.95a	0.69b^∗^	0.80b^∗^	0.95a	0.97a	0.95a
	HHZ	0.77b^∗^	0.63b^∗^	0.69c^∗^	0.97a	0.92a	0.96a
	LYPJ	0.78b^∗^	0.71b^∗^	0.74bc^∗^	0.99a	0.91a	0.97a
	SY63	1.02a	0.85a^∗^	0.90a^∗^	0.98a	0.97a	0.98a
HDT	N22	0.88b^∗^	0.77a^∗^	0.80b^∗^	0.97a	0.93a	0.95a
	HHZ	0.84bc^∗^	0.34c^∗^	0.57c^∗^	0.96a	0.94a	0.95a
	LYPJ	0.73c^∗^	0.59b^∗^	0.65c^∗^	0.87b^∗^	0.87a^∗^	0.87b^∗^
	SY63	1.00a	0.85a^∗^	0.90a^∗^	0.98a	0.89a	0.96a
ADT	N22	0.71b^∗^	0.32b^∗^	0.45b^∗^	0.96a	0.90a	0.92ab
	HHZ	0.67b^∗^	0.27b^∗^	0.45b^∗^	0.94a	0.89a	0.93a
	LYPJ	0.61b^∗^	0.30b^∗^	0.43b^∗^	0.77b^∗^	0.81a^∗^	0.78b^∗^
	SY63	1.00a	0.83a^∗^	0.84a^∗^	0.96a	0.91a	0.93a

*Relative values were presented in the table. A relative value was defined as the ratio of the score under a high temperature treatment to that under the control treatment for the same trait in the same variety. Different letters indicate significant differences among varieties under the same temperature treatment by a least significant difference (LSD) test at *P* < 0.05. A relative value less or more than 1.0 indicated a decrease or increase in a trait under a high temperature treatment compared with that under the control condition, respectively. Asterisks indicate significant differences when comparing the absolute mean of a trait under a given high temperature treatment with that under the control by LSD test at *P* < 0.05.*

Generally, the three high temperature treatments had no effects on panicle tZ+tZR content in the SY63 variety, which showed the highest relative abundance of panicle CTKs among the four tested varieties (**Table 1**).

### CTK Abundance in Roots under High Temperature Treatments

The absolute concentrations of tZ+tZR, iPMP+iP+iPA, and aCTKs in roots were 364, 232, and 596 ng/g in N22, 726, 201, and 926 ng/g in HHZ, 748, 253, and 1001 ng/g in LYPJ, and 622, 193, and 815 ng/g in SY63 under control temperature treatment, respectively (Supplementary Table S1).

There was no significant reduction in the abundance of tZ+tZR, iPMP+iP+iPA, or aCTKs in the roots of the N22, HHZ, and SY63 varieties under the three high temperature treatments. However, in the LYPJ variety, the abundance of tZ+tZR, iPMP+iP+iPA, and aCTKs was reduced significantly by the HDT and ADT treatments (**Table 1**).

### Xylem Sap CTK Content and CTK Transport Rate under High Temperature Treatments

The absolute rates of xylem sap flow were 86, 151, 143, and 85 mg/tiller/h in N22, HHZ, LYPJ, and SY63 under control temperature treatment, respectively (Supplementary Table S2).

The high temperature treatments significantly reduced xylem sap flow in the HHZ, LYPJ, and N22 varieties; xylem sap flow was reduced, on average, by 17% (9–23%) in HHZ, 21% (8–27%) in LYPJ, and 19% (8–27%) in the N22 variety across the three high temperature treatments. However, the xylem sap flow of the SY63 variety was significantly increased, on average, by 18% (11–22%) across the three high temperature treatments. The relative xylem sap flow in the SY63 variety was significantly higher than that of the HHZ, LYPJ, and N22 varieties (**Table 2**).

**Table 2:** Relative CTK concentrations and transport rates of CTKs in xylem sap under high temperature treatments.

Treatment	Variety	Xylem sap flow	CTKs in xylem sap			Transport rate of CTKs via xylem sap		
			tZ+tZR	iPMP+iP+iPA	aCTKs	tZ+tZR	iPMP+iP+iPA	aCTKs
HNT	N22	0.92b^∗^	0.97a	0.96a	0.97a	0.92b	0.9lab	0.92b
	HHZ	0.83b^∗^	0.93a	0.85a*	0.98a	0.85b*	0.70b*	0.83b*
	LYPJ	0.91b^∗^	0.96a	0.94a	0.95a	0.87b*	0.85b*	0.87b*
	SY63	1.21a^∗^	0.98a	0.93a	0.97a	1.22a*	1.14a	1.21a*
HDT	N22	0.73c^∗^	0.91a	0.96a	0.91a	0.66b*	0.69b*	0.67b*
	HHZ	0.91b^∗^	0.95a	0.85b*	0.93a	0.86b*	0.78b*	0.85b*
	LYPJ	0.73c^∗^	0.91a	0.82b*	0.90a	0.66b*	0.59b*	0.65b*
	SY63	1.21a^∗^	0.95a	0.95a	0.95a	1.25a*	1.21a	1.24a*
ADT	N22	0.77b^∗^	0.87a^∗^	0.82ab*	0.89a*	0.67b*	0.62b*	0.66b*
	HHZ	0.75b^∗^	0.83a^∗^	0.78b*	0.79b*	0.61b*	0.59b*	0.61b*
	LYPJ	0.75b^∗^	0.91a	0.72b*	0.89a*	0.68b*	0.54b*	0.66b*
	SY63	1.11a^∗^	0.95a	0.91a	0.95a	1.05a	1.05a	1.05a

*A relative value was defined as the ratio of the score under a high temperature treatment to that under the control treatment for the same trait in the same variety. Different letters indicate significant differences among varieties under the same temperature treatment by a LSD test at *P* < 0.05. A relative value less or more than 1.0 indicated a decrease or increase in a trait under a high temperature treatment compared with that under the control condition, respectively. Asterisks indicate significant differences when comparing the absolute mean of a trait under a given high temperature treatment with that under the control by LSD test at *P* < 0.05.*

The absolute concentrations of tZ+tZR, iPMP+iP+iPA, and aCTKs in xylem sap were 1.30, 0.19, and 1.49 pg/ml in N22, 1.70, 0.26, and 1.96 pg/ml in HHZ, 1.66, 0.26, and 1.92 pg/ml in LYPJ, and 1.56, 0.22, and 1.78 pg/ml in SY63 under control temperature treatment, respectively (Supplementary Table S2).

The HNT treatment did not substantially decrease the abundance of xylem sap CTKs in the four varieties. Similar effects were observed under the HDT treatment, but iP-type CTKs were reduced in abundance in the HHZ and LYPJ varieties. The three temperature treatments had no effects on tZ+tZR; however, the high temperature treatments decreased the abundance of iP-type CTKs in the N22, HHZ, and LYPJ varieties, as well as the abundance of aCTKs in the HHZ and LYPJ varieties. Generally, the three high temperature treatments imposed small effects on CTK concentrations in xylem sap; most of the effects of high temperature were not significant, with the exception of the significant effects of the ADT treatment on the LYPJ, HHZ, and N22 varieties (**Table 2** and Supplementary Table S2).

The absolute transport rates of tZ+tZR, iPMP+iP+iPA, and aCTKs were 0.11, 0.02, and 0.13 pg/tiller/h in N22, 0.26, 0.04, and 0.30 pg/tiller/h in HHZ, 0.24, 0.04, and 0.27 pg/tiller/h in LYPJ, and 0.13, 0.02, and 0.15 pg/tiller/h in SY63 under control temperature treatment, respectively (Supplementary Table S2).

The transport rate of CTKs changed in a manner similar to that of xylem sap flow in the four rice varieties (**Table 2** and Supplementary Table S2). The transport rates of tZ+tZR, iPMP+iP+iPA, and aCTKs were reduced significantly in the HHZ, LYPJ, and N22 varieties in response to the three high temperature treatments, while these transport rates were increased or not affected by the treatments in the SY63 variety. The relative transport rate of CTKs in xylem sap in the SY63 variety was significantly greater than the corresponding rates in the HHZ, LYPJ, and N22 varieties.

### Activity Levels of CKX, LOG, IPT, and CYP735A under High Temperature Treatments

Absolute activity levels of CKX were 22.0, 8.0, 17.9, and 23.8 nmol iP/mg protein/h in N22, HHZ, LYPJ, and SY63 under control temperature, respectively (Supplementary Table S3). The three high temperature treatments significantly increased the activity level of CKX in the HHZ, LYPJ, and N22 varieties; however, the treatments had no substantial effects in the SY63 variety (**Table 3** and Supplementary Table S3). Additionally, the relative activity level of CKX in the SY63 variety was significantly lower than that of the HHZ, LYPJ, and N22 varieties (**Table 3**).

**Table 3:** Relative activity levels of CKX, IPT, LOG, and CYP735A under high temperature treatments.

Treatment	Variety	CKX	IPT	LOG	CYP735A
HNT	N22	1.84a*	0.79b*	0.87a	0.80a*
	HHZ	2.14a*	0.90a	0.85a*	0.82a*
	LYPJ	1.97a*	0.91a	0.86a	0.64b*
	SY63	1.06b	0.94a	0.87a	0.87a
HDT	N22	2.00a*	0.72a*	0.58b*	0.82a*
	HHZ	1.36b*	0.49b*	0.60b*	0.62b*
	LYPJ	1.62b*	0.51b*	0.71a*	0.73ab*
	SY63	1.04c	0.66a*	0.60b*	0.76a*
ADT	N22	2.57a*	0.62a*	0.60a*	0.74a*
	HHZ	2.53a*	0.50b*	0.52a*	0.61b*
	LYPJ	2.22a*	0.39c*	0.58a*	0.74a*
	SY63	1.18b	0.55ab*	0.55a*	0.72a*

*Relative values were presented in the table. A relative value was defined as the ratio of the score under a high temperature treatment to that under the control treatment for the same trait in the same variety. Different letters indicate significant differences among varieties under the same temperature treatment by a LSD test at *P* < 0.05. A relative value less or more than 1.0 indicated a decrease or increase in a trait under a high temperature treatment compared with that under the control condition, respectively. Asterisks indicate significant differences when comparing the absolute mean of a trait under a given high temperature treatment with that under the control by LSD test at *P* < 0.05.*

Absolute activity levels of IPT, LOG, and CYP735A were 0.17 nmol iPMP/mg protein/h, 1.63 nmol iP/mg protein/h, and 0.22 nmol tZR/mg protein/h in N22, 0.04, 1.93, and 0.13 in HHZ, 0.11, 1.65, and 0.28 in LYPJ, and 0.09, 1.67, and 0.23 in SY63, respectively (Supplementary Table S3).

Generally, the activity levels of IPT, LOG, and CYP735A were slightly or significantly reduced by HNT in the four tested rice varieties; however, the activity levels of IPT, LOG, and CYP735A were reduced significantly under the HDT and ADT treatments. The relative activity levels of IPT, LOG, and CYP735A under the HNT treatment were higher than their activity levels under the HDT and ADT treatments. Additionally, the SY63 and N22 varieties generally showed high relative activity levels of IPT, LOG, and CYP735A in comparison with those of the HHZ and LYPJ varieties (**Table 3**).

### Relationships of Panicle CTKs with Its Transportation via Xylem Sap

**FIGURE 3:**
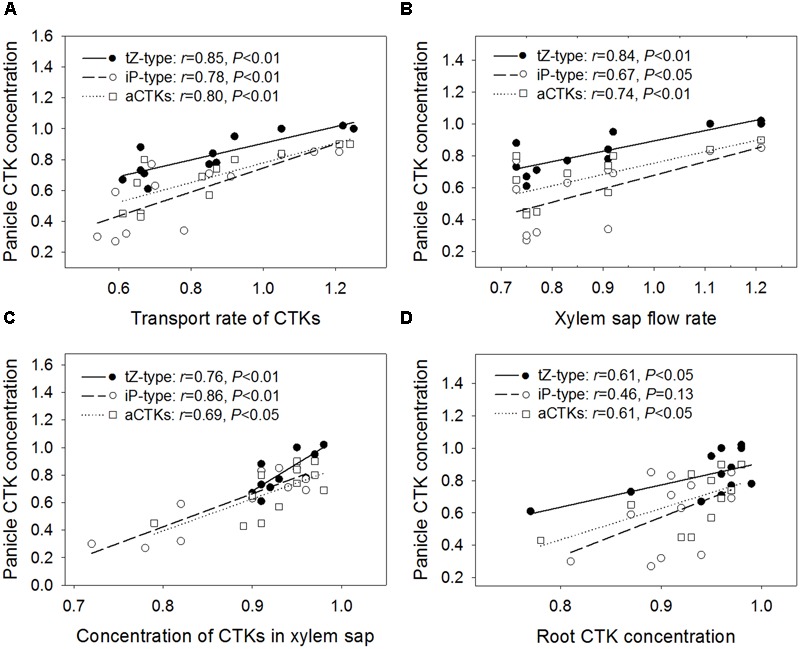
**Regression analysis for the relative transport rate of CTKs (A)**, relative xylem sap flow rate **(B)**, relative root xylem sap CTK concentration **(C)**, and relative root CTK concentration **(D)** with relative panicle CTK concentration across four varieties and three high temperature treatments.

As shown in **Figure 3A**, the relative concentrations of tZ-type CTKs, iP-type CTKs, and aCTKs in panicles were positively and significantly correlated with their relative transport rates via xylem sap. It was also observed that concentrations of panicle CTKs were significantly and positively correlated with the xylem sap flow rate (**Figure 3B**) and CTK concentrations in xylem sap (**Figure 3C**). Additionally, panicle CTK concentrations showed positive correlations with root CTK concentrations, with the exception of iP-type CTKs (**Figure 3D**).

Generally, CTK concentrations in xylem sap were not correlated significantly with root CTK concentrations, with the exception of iP-type CTKs (*r* = 0.77, *P* < 0.01 for tZ-type; *r* = 0.74, *P* < 0.01 for iP-type; *r* = 0.80, *P* < 0.01 for aCTKs, *n* = 12). The CTK transport rate via xylem sap was poorly correlated with root CTK concentrations, with the exception of iP-type CTKs (*r* = 0.56, *P* = 0.06 for tZ-type; *r* = 0.51, *P* = 0.09 for iP-type; *r* = 0.59, *P* < 0.05 for aCTKs, *n* = 12).

### Relationships between Panicle CTK Abundance and CTK Metabolism-Related Enzymes

**FIGURE 4:**
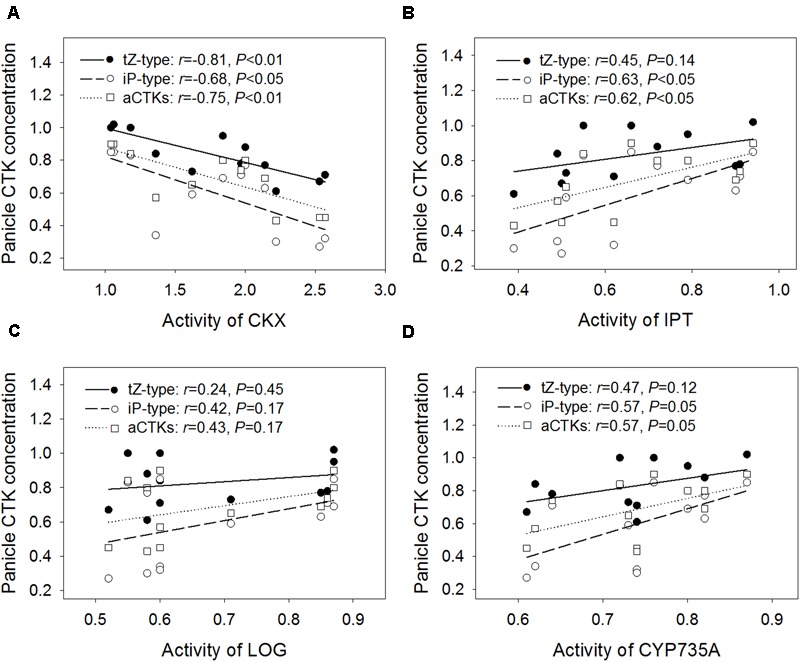
**Regression analysis for the relative activity levels of CKX (A)**, IPT **(B)**, LOG **(C)**, and CYP735A **(D)** with the relative concentration of panicle CTKs across four varieties and three high temperature treatments.

The relative contents of tZ-type CTKs, iP-type CTKs, and aCTKs in panicles were significantly and negatively correlated with the relative CKX activity level in panicles (*r* = -0.81 for tZ-type, -0.68 for iP-type, and -0.75 for aCTKs) (**Figure 4A**). The relative contents of iP-type CTKs and aCTKs were significantly and positively correlated with the relative activities of IPT (*r* = 0.63 for iP-type and 0.62 for aCTKs, **Figure 4B**), and CYP735A (*r* = 0.57 for iP-type, and 0.57 for aCTKs, **Figure 4D**); however, tZ-type CTK abundance was poorly correlated with the activity levels of IPT, LOG, and CYP735A.

### Relationships of the Number of Spikelets per Panicle with CTK Content and Metabolism-Related Enzyme Activity Levels

**FIGURE 5:**
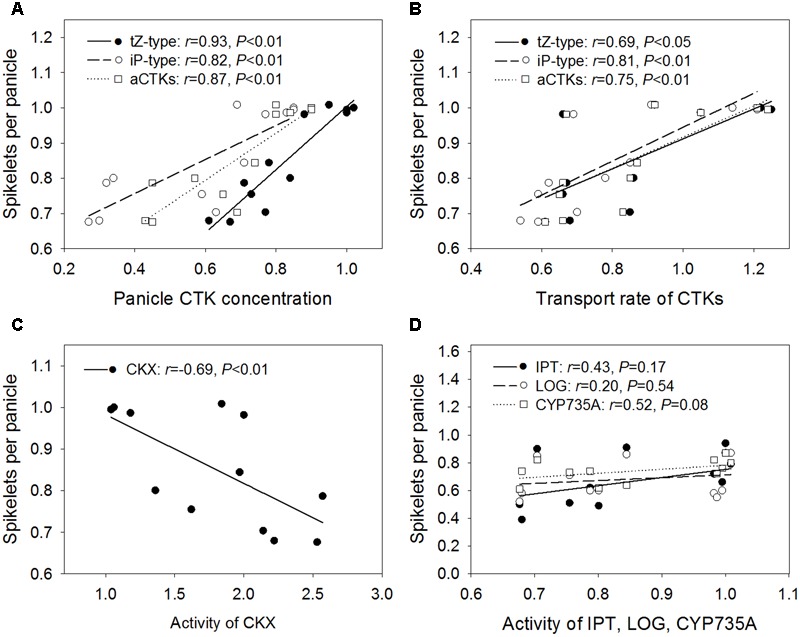
**Regression analysis for the relative concentrations of panicle CTKs (A)**, relative transport rate of xylem CTKs **(B)**, and relative activity levels of CKX **(C)**, IPT, LOG, and CYP735A **(D)** with the relative number of spikelets per panicle across four varieties and three high temperature treatments.

The relative number of spikelets per panicle was positively and significantly correlated with the relative concentrations of tZ-type CTKs (*r* = 0.93), iP-type CTKs (*r* = 0.82), and aCTKs (*r* = 0.87) in the panicles (**Figure 5A**), as well as with the relative transport rates of tZ-type CTKs (*r* = 0.69), iP-type CTKs (*r* = 0.81), and aCTKs (*r* = 0.75) (**Figure 5B**). The relative number of spikelets per panicle was negatively and significantly correlated with the relative activity level of CKX (*r* = -0.69, **Figure 5C**). The relative activity levels of IPT, LOG, and CYP735A were poorly correlated with the relative number of spikelets per panicle (**Figure 5D**).

## Discussion

### Response of Panicle Size to High Temperature in Rice Varieties

Panicle size was reduced in response to the three high temperature treatments, especially in the HHZ, LYPJ, and N22 varieties (**Figure 2**). Previous studies also reported reduced panicle size in rice plants subjected to high temperature conditions (Wei et al., 2010; Wang et al., 2015). Heat-induced reduction in panicle size was associated with disruption of differentiation and induction of degradation of secondary branches and attached florets (Wang et al., 2015). The SY63 variety showed relatively stable panicle size under the three high temperature treatments. Moreover, the relative number of spikelets per SY63 panicle was significantly higher than that of the panicles of the HHZ and LYPJ varieties under the HNT, HDT, and ADT treatments (**Figure 2**). Therefore, the results presented in this study show that the effect of high temperature on panicle size is dependent on the variety of rice subjected to high temperature conditions. In addition, the SY63 variety has heat tolerance greater than that of the HHZ variety or LYPJ variety. Similarly, Wang et al. (2015) also reported genotypic variation in the effect of high temperature on panicle size during the early reproductive phase in rice. It is noteworthy that the N22 variety showed heat tolerance under the HNT and HDT conditions (**Figure 2**). However, the N22 variety suffered from heat stress injury under the ADT treatment (a combination of the HNT and HDT treatments), in contrast to the SY63 variety and similar to the heat-susceptible HHZ and LYPJ varieties (**Figure 2**). The increased intensity of high temperature conditions aggravates injury in rice during the reproductive stage (Jagadish et al., 2007). Therefore, our data suggest that the N22 variety can likely withstand only low intensity of heat stress, whereas the SY63 variety may be able to withstand high intensity of heat stress (a combination of the HNT and HDT treatments).

### Response of Panicle CTKs to High Temperature and Relationship with Panicle Size

Panicle CTK abundance was reduced in response to high temperature during the early reproductive stage, especially in the heat-susceptible LYPJ and HHZ varieties (**Table 1**). This result was in agreement with previous observations that high temperature reduced the abundance of active CTKs (aCTKs) in *Arabidopsis* and *Phalaenopsis* (Chou et al., 2000; Skalák et al., 2016).

**FIGURE 6:**
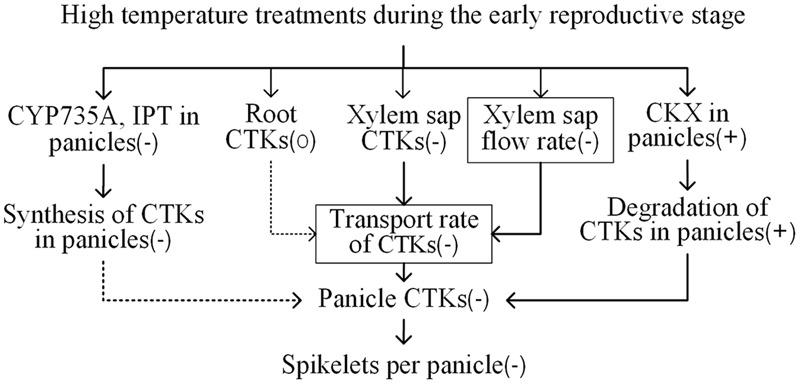
**Proposed mechanism by which cytokinins mediate changes in the number of spikelets per panicle in response to high temperature treatments during the early reproductive phase in rice.** -, +, and 0 indicate a decrease, an increase, and relative stability in a certain trait under high temperature treatment, respectively. → indicates a significant correlation between two traits. ⤑ indicates no significant correlation between two traits. The value of the trait with a box was decreased in heat-susceptible varieties while increased in heat-tolerant variety SY63 under the three high temperature treatments. The other traits without a box showed similar responses to the high temperature treatments, which showed larger changes in heat-susceptible varieties than that in heat-tolerant variety.

Under the HNT and HDT treatments, the N22 variety, which showed a stable number of spikelets per panicle, had relatively stable/high aCTK abundance in panicles; however, the N22 variety showed large reductions in the number of spikelets per panicle and the abundance of iP-type CTKs and aCTKs under the ADT treatment (combination of the HDT and HNT treatments), and the relative aCTK abundance of the N22 variety under the ADT treatment was similar to that of the heat-susceptible HHZ and LYPJ varieties (**Table 1**). In tobacco leaves, the abundance of aCTKs was reduced continuously as the duration of high temperature treatment was prolonged (Macková et al., 2013). However, in heat-tolerant variety SY63, which had a stable number of spikelets per panicle, the abundance of panicle tZ-type CTKs was not affected by the three high temperature treatments. Although panicle iP-type CTKs and aCTKs were reduced significantly in abundance by the high temperature treatments in the SY63 variety, their abundance remained relatively stable in comparison with that of the same CTKs in the other three varieties (**Table 1**). Heat-induced reduction in the number of spikelets per panicle was alleviated by application of exogenous BAP to heat-susceptible variety Liangyoupeijiu, which showed reduced CTK abundance under the high temperature treatments (**Figure 1**). In rice, exogenous BAP application increased the abundance of tZ-type CTKs (Liu et al., 2011) and the number of spikelets per panicle (Ding et al., 2014). Similarly, several passion fruit species and *Arabidopsis* ecotypes that showed stable abundance of aCTKs had less severe reductions in floret number in comparison with species and ecotypes that showed reduced CTK abundance in response to high temperature when exogenous CTKs were applied to mitigate the effect of heat injury on floret growth (Sobol et al., 2014). Additionally, we observed that relative panicle CTK concentrations were positively correlated with the relative number of spikelets per panicle (**Figure 5A**). In our study, reductions in the number of spikelets per panicle were associated with reduced panicle CTK abundance under high temperature treatments (**Figure 6**). The stability of panicle CTKs may be involved in maintaining panicle size under high temperature conditions in heat-tolerant variety SY63.

This study assessed the stability of tZ-type CTKs in SY63 panicles under high temperature treatments (**Table 1**). tZ-type CTKs are thought to act as messengers from root to shoot, while iP-type CTKs act as messengers from shoot to shoot (Kudo et al., 2010). Root-synthesized CTKs are transported to the aerial organs via xylem sap flow (Mader et al., 2003; Aloni et al., 2005; Yong et al., 2014). In SY63 variety, stable tZ-type CTKs in panicles may be partially attributed to stable import of root CTKs via enhanced xylem sap flow under the high temperature treatments (**Table 2**).

### CTK Translocation and Its Relationship with Panicle Size under High Temperature Treatments

We observed that xylem sap flow showed genotypic variation in response to high temperature. In heat-susceptible varieties, xylem sap flow was decreased significantly by exposure to high temperature, while heat-tolerant variety SY63 showed significantly increased xylem sap flow after 15 days of high temperature treatment during the early reproductive phase (**Table 2**). Tao et al. (2009) found that heat-tolerant rice varieties showed enhanced exudation of xylem sap during 5 weeks of high temperature treatment; however, xylem sap exudation was inhibited significantly after 2 weeks of high temperature treatment in heat-susceptible rice varieties. Xylem sap flow is closely associated with transpiration flow (Boonman et al., 2007; Kudo et al., 2010). Under high temperature stress, the increased transpiration flow from roots may be importance for the decrease in leaf surface temperature via evaporative cooling, and for transport of root-derived messengers (such as ABA) transport to shoot to relief heat injury (Zandalinas et al., 2016). Therefore, our results suggest that heat-tolerant rice varieties may relieve heat stress via persistent enhancement of xylem sap flow in comparison that of heat-sensitive varieties.

In the present study, root CTKs and xylem sap CTKs showed similar responses to high temperature treatments; their abundance was slightly reduced in comparison with that of panicle CTKs (**Tables 1, 2**), indicating that the effects of high temperature treatments on panicle CTKs were more severe than their effects on root CTKs. Similarly, Takei et al. (2001b) reported that root CTKs showed changes in abundance that corresponded with changes in CTKs transport from root to shoot via xylem sap. In apple trees, exposure of roots to high temperature treatment produced negligible effects on xylem sap CTKs (Tromp and Ovaa, 1994). Additionally, high air temperature produced minor effects on CTKs in roots, which were less severe than the effects of high air temperature on CTKs in the aerial organs of pea plants (Vaseva et al., 2009). These studies demonstrate that CTKs in roots and xylem sap were less sensitive than CTKs in aerial organs, such as panicles, to high temperature.

In this study, the transport rate of CTKs changed in response to high temperature in a manner similar to that of xylem sap flow (*r* = 0.98, *P* < 0.01, *n* = 12). The transport rate of CTKs was decreased significantly by high temperature treatments in heat-susceptible varieties (LYPJ, HHZ, and N22), which showed decreased xylem sap flow rate in comparison with that of heat-tolerant variety SY63. However, the transport rate of CTKs in heat-tolerant variety SY63 increased as the xylem sap flow rate increased (**Table 2**). CTK transport is associated with xylem sap flow, which is closely associated with transpiration flow (Boonman et al., 2007; Kudo et al., 2010). In shade, reduced CTK transport leads to decreased CTK abundance in aerial organs (Boonman et al., 2007). In our study, the relative transport rate of CTKs was significantly correlated with relative CTK abundance in young panicles (**Figure 3**) and the relative number of spikelets per panicle (**Figure 5**). Root-derived CTKs translocated via xylem sap mediate apical shoot development (Aloni et al., 2005). Changes in xylem sap CTK abundance in response to environmental conditions regulate plant adaptation to adverse stresses (Ambler et al., 1992; Takei et al., 2001b; Alvarez et al., 2008; Kudoyarova et al., 2014). Taken together, our results suggest that transport of CTKs via xylem sap influences the number of spikelets per panicle by adjusting panicle CTK abundance under high temperature conditions (**Figure 6**). A relatively high CTK transport rate and relatively good panicle CTK stability likely contribute to the minimal reduction in the number of spikelets per panicle observed in heat-tolerant variety SY63 in response to high temperature.

### Response of Enzymes Involved in CTK Metabolism to High Temperature and Their Relationship with Panicle Size

In this study, CTK metabolism-related enzymes CKX, IPT, LOG, and CYP735A were regulated to different extents by high temperature treatments in a manner dependent on rice variety. The activity level of CKX increased significantly in heat-susceptible varieties LYPJ, HHZ, and N22; however, heat-tolerant variety SY63 showed no substantial changes in CKX activity under any of the three tested high temperature treatments. The activity levels of LOG, IPT, and CYP735A were reduced by high temperature in all four tested varieties (**Table 3**). Similar results were obtained in previous studies, in which the activity level of CKX was increased in *Nicotian*a *tabacum* and *Pisum sativum* under high temperature conditions (Vaseva-Gemisheva et al., 2004; Lubovská et al., 2014), whereas the activity levels of IPT, CYP735A, and LOG were suppressed by various abiotic stresses such as heat, cold, and drought (Maruyama et al., 2014; Skalák et al., 2016). These results suggest that stress conditions, including heat stress, disrupt the balance between synthesis and catabolism of CTKs via regulation of the activity levels of related enzymes.

Most previous studies have separately assessed the roles of IPT (Ding et al., 2014), CYP735A (Takei et al., 2004), LOG (Kurakawa et al., 2007), and CKX (Ashikari et al., 2005) in regulating CTK abundance and panicle size in rice and *Arabidopsis*; however, it is not yet clear which processes or enzymes play important roles in heat tolerance. CKX activity was stable under each high temperature treatment in heat-tolerant variety SY63, which showed stable panicle CTK abundance and a consistent number of spikelets per panicle. Correlation analysis also revealed that the relative CKX activity level was significantly and negatively correlated with relative panicle CTK abundance and the relative number of spikelets per panicle (**Figures 4A, 5C**). In rice and *Nicotiana*, CKX is involved in the effect of high temperature on plant growth (Tripathi et al., 2012; Lubovská et al., 2014). Although certain, but not always significant, relationships were found between the activity levels of CTK biosynthetic enzymes (IPT and CYP735A) and panicle CTK abundance (**Figure 4**), none of these enzymes had an activity level that was correlated with the relative number of spikelets per panicle (**Figure 5D**). However, it is noteworthy that the activity levels of IPT, LOG, and CYP735A were reduced significantly by the HDT and ADT treatments. Moreover, slight correlations were found between the activity levels of IPT and CYP735A and the concentrations of iP-type CTK and aCTK in panicles (**Figures 4B,D**). Therefore, the relationship between reductions in the activity levels of IPT and CYP735A and panicle size merit further investigation. Our study demonstrates that CKX activity seems to influence panicle size under high temperature treatments by regulating panicle CTK abundance (**Figure 6**). The stable CKX activity of heat-tolerant variety SY63 may underlie its stable panicle CTK abundance and consistent number of spikelets per panicle under high temperature conditions.

## Conclusion

The three high temperature treatments significantly decreased the number of spikelets per panicle in heat-susceptible rice varieties (HHZ, LYPJ, and N22), whereas heat-tolerant variety SY63 showed a relatively stable number of spikelets per panicle. Application of exogenous BAP increased the number of spikelets per panicle in the LYPJ variety under the ADT treatment, indicating that CTKs are involved in panicle differentiation under high temperature conditions.

The three high temperature treatments significantly decreased panicle CTK abundance, xylem sap flow rate, and the transport rate of CTKs via xylem sap flow, whereas the treatments increased the activity level of CKX in three heat-sensitive rice varieties (HHZ, LYPJ, and N22). In comparison with the heat-susceptible varieties, heat-tolerant variety SY63 showed more stable panicle CTK abundance, an enhanced xylem sap flow rate, a greater CTK transport rate, and more stable CKX activity under the three high temperature treatments. The activity levels of enzymes involved in CTK synthesis (IPT, LOG, and CYP735A) were decreased by the high temperature treatments, especially by the HDT and ADT treatments.

Generally, panicle CTK concentrations were significantly and positively correlated with the xylem sap flow rate, transport rate of CTK via xylem sap, and CTK concentrations in xylem sap and roots, whereas panicle CTK concentrations were negatively correlated with CKX activity and slightly and positively correlated with the activity levels of IPT, and CYP735A.

According to our results, reduced panicle CTK abundance in heat-susceptible rice varieties under the high temperature treatments was associated with: (i) the reduced transport rate of xylem sap CTKs, which was resulted from the reduced xylem sap flow and the decreased xylem sap CTK concentration; (ii) increased activity of CKX in young panicles, which promoted degradation of panicle CTKs; (iii) decreased activity levels of IPT, and CYP735A, regardless of the insignificant correlations between the relative activity levels of these enzymes and relative panicle size. Therefore, CTK transport from root to shoot and CTK degradation via CKX are the key processes that determine panicle CTK abundance and thus regulate panicle size under high temperature conditions.

Generally, stable CTK concentrations under high temperature conditions inhibited the reduction in the number of spikelets per panicle in heat-tolerant variety SY63 in comparison with that which occurred in the heat-susceptible varieties; this effect was attributed to enhanced transportation of root-derived CTKs due mainly to strong xylem sap flow and impaired CTK degradation in panicles as a result of a relatively low level of panicle CKX activity. Therefore, a high transport rate of CTKs and low CKX activity are required to stabilize panicle size under heat stress (**Figure 6**). From the viewpoint of plant hormone metabolism, our results provide insight into the mechanisms underlying rice heat tolerance, laying a foundation for effective breeding and selection of rice varieties with high temperature tolerance and maximal grain yield.

## Author Contributions

CW and KC designed the experiments. CW, QL, WW, SF, and QH performed the experiments, KC, JH, LN, and SP performed parts of the experiments. CW and KC analyzed the data and wrote the manuscript. SF, JH, LN, PM, and SP revised the manuscript. All authors have read and approved the final manuscript.

## Conflict of Interest Statement

The authors declare that the research was conducted in the absence of any commercial or financial relationships that could be construed as a potential conflict of interest.
