# Supplementary material for: Heat-Induced Cytokinin Transportation and Degradation Are Associated with Reduced Panicle Cytokinin Expression and Fewer Spikelets per Panicle in Rice
Source: Front Plant Sci. 2017 Mar 17;8:371. doi: 10.3389/fpls.2017.00371 (PMC5355447; doi:10.3389/fpls.2017.00371)
Supplement: Supplementary file 1 [file Table_1.DOC]

*Supplementary Material*

Heat-induced cytokinin transportation and degradation are associated with reduced panicle cytokinin expression and fewer spikelets per panicle in rice

**Authors: Chao Wu^1^, Kehui Cui^1*^, Wencheng Wang^1^, Qian Li^1^, Shah Fahad^1^, Qiuqian Hu^1^, Jianliang Huang^1^, Lixiao Nie^1^, Pravat K. Mohapatra^2^ and Shaobing Peng^1^**

**Institution addresses:**

^1^National Key Laboratory of Crop Genetic Improvement, MOA Key Laboratory of Crop Ecophysiology and Farming System in the Middle Reaches of the Yangtze River, Hubei Collaborative Innovation for Grain Industry, Huazhong Agricultural University, Wuhan, Hubei 430070, China,

^2^School of Life Sciences, Sambalpur University, Sambalpur, Odisha, India

* **Corresponding author:** Kehui Cui

E-mail: [cuikehui@mail.hzau.edu.cn](mailto:cuikehui@mail.hzau.edu.cn)

fax: +0086-27-87288380

tel: +0086-27-87288380

**Table S1**. CTK concentrations in the panicles and roots under high temperature

| Treatment | Variety | CTKs in the panicle (ng/g) | | | CTKs in the root (ng/g) | | |
| --- | --- | --- | --- | --- | --- | --- | --- |
|  |  | tZ+ tZR | iPMP+ iP+ iPA | aCTKs | tZ+ tZR | iPMP+ iP+ iPA | aCTKs |
| N22 | CK | 114a | 223a | 336a | 364a | 232a | 596a |
|  | HNT | 116a | 152c | 268b | 342a | 229a | 571a |
|  | HDT | 100b | 170b | 270b | 351a | 210ab | 561a |
|  | ADT | 80c | 72d | 152c | 345a | 193b | 538a |
| HHZ | CK | 169a | 208a | 376a | 726a | 201a | 926a |
|  | HNT | 129b | 131b | 260b | 704a | 184a | 888a |
|  | HDT | 142b | 71c | 213c | 693a | 189a | 882a |
|  | ADT | 112c | 56d | 168d | 670a | 177a | 848a |
| LYPJ | CK | 184a | 257a | 441a | 748a | 253a | 1001a |
|  | HNT | 145b | 184b | 329b | 730a | 231ab | 961a |
|  | HDT | 135b | 150c | 285c | 652b | 220b | 872b |
|  | ADT | 112c | 77d | 189d | 589b | 204b | 793b |
| SY63 | CK | 114a | 238a | 353a | 622a | 193a | 815a |
|  | HNT | 116a | 201b | 318b | 608a | 187a | 795a |
|  | HDT | 114a | 201b | 316b | 600a | 170a | 770a |
|  | ADT | 111a | 182b | 296b | 591a | 167a | 758a |

Data are presented as mean ± SD (*n* = 4). Different letters within a column indicate statistical differences among the four temperature treatments for each variety at the *P* < 0.05 level by the least significant difference (LSD) test.

aCTKs, active cytokinin compounds (tZ+tZR+iP+iPA+iPMP); ADT, high whole-day temperature treatment; CK, control; CTKs, total cytokinins; HDT, high daytime temperature treatment; HHZ, Huanghuazhan; HNT, high nighttime temperature treatment; LYPJ, Liangyoupeijiu; N22, Nagina22; SY63, Shanyou 63. Data of panicle CTKs were published in our previous study (Heat-induced phytohormone changes are associated with disrupted early reproductive development and reduced yield in rice. Sci. Rep., 2016. doi: 10.1038/srep34978)

**Table S2**. CTK concentrations and transport rate of CTKs in xylem sap under high temperature

| Treatment | Variety | Xylem sap rate(mg/tiller/hr) | CTKs in xylem sap (pg/ml) | | | Transport rate of CTKs (pg/tiller/hr) | | |
| --- | --- | --- | --- | --- | --- | --- | --- | --- |
|  |  |  | tZ+ tZR | iPMP+ iP+ iPA | aCTKs | tZ+ tZR | iPMP+ iP+ iPA | aCTKs |
| N22 | CK | 86a | 1.30a | 0.19a | 1.49a | 0.11a | 0.02a | 0.13a |
|  | HNT | 79b | 1.26ab | 0.18a | 1.44ab | 0.10a | 0.01a | 0.12a |
|  | HDT | 63b | 1.17ab | 0.18a | 1.35ab | 0.07b | 0.01b | 0.09b |
|  | ADT | 66b | 1.14b | 0.15b | 1.29b | 0.08b | 0.01b | 0.09b |
| HHZ | CK | 151a | 1.70a | 0.26a | 1.96a | 0.26a | 0.04a | 0.30a |
|  | HNT | 123b | 1.70a | 0.22b | 1.92a | 0.21b | 0.03bc | 0.24b |
|  | HDT | 137b | 1.61a | 0.22b | 1.82a | 0.22b | 0.03b | 0.25b |
|  | ADT | 117b | 1.37b | 0.20b | 1.57b | 0.16c | 0.02c | 0.18c |
| LYPJ | CK | 143a | 1.66a | 0.26a | 1.92a | 0.24a | 0.04a | 0.27a |
|  | HNT | 131b | 1.64a | 0.25a | 1.89ab | 0.22b | 0.03b | 0.25b |
|  | HDT | 104c | 1.56a | 0.21b | 1.78ab | 0.16c | 0.02c | 0.19c |
|  | ADT | 106c | 1.50a | 0.18c | 1.69b | 0.16c | 0.02c | 0.18c |
| SY63 | CK | 85b | 1.56a | 0.22a | 1.78a | 0.13b | 0.02a | 0.15b |
|  | HNT | 103a | 1.52a | 0.20a | 1.72a | 0.16a | 0.02a | 0.18a |
|  | HDT | 104a | 1.48a | 0.21a | 1.69a | 0.15a | 0.02a | 0.18a |
|  | ADT | 95a | 1.49a | 0.20a | 1.69a | 0.14ab | 0.02a | 0.16ab |

Data are presented as mean ± SD (*n* = 4). 1 g xylem sap is presumably equal to 1 ml. Different letters within a column indicate statistical differences among the four temperature treatments for each variety at the *P* < 0.05 level by the least significant difference (LSD) test.

aCTKs, active cytokinin compounds (tZ+tZR+iP+iPA+iPMP); ADT, high whole-day temperature treatment; CK, control; CTKs, total cytokinins; HDT, high daytime temperature treatment; HHZ, Huanghuazhan; HNT, high nighttime temperature treatment; LYPJ, Liangyoupeijiu; N22, Nagina22; SY63, Shanyou 63.

**Table S3**. Activity of CKX, IPT, LOG and CYP735A in panicles under high temperature

| Variety | Treatment | CKX  (nmol iP/mg Protein /hr) | IPT  (nmol iPMP/mg Protein /hr) | LOG  (nmol iP/mg Protein/hr) | CYP  (nmol tZR/mg Protein /hr) |
| --- | --- | --- | --- | --- | --- |
| N22 | CK | 22.0d | 0.17a | 1.63a | 0.22a |
|  | HNT | 37.7c | 0.13b | 1.46ab | 0.18b |
|  | HDT | 48.0b | 0.13b | 1.03b | 0.18b |
|  | ADT | 69.7a | 0.12b | 1.08b | 0.16b |
| HHZ | CK | 8.0c | 0.04a | 1.73a | 0.13a |
|  | HNT | 16.2a | 0.04a | 1.45b | 0.11b |
|  | HDT | 11.0b | 0.02b | 1.01c | 0.08b |
|  | ADT | 18.1a | 0.02b | 0.87c | 0.08b |
| LYPJ | CK | 17.9c | 0.11a | 1.54a | 0.28a |
|  | HNT | 37.9a | 0.11a | 1.30ab | 0.17b |
|  | HDT | 28.2b | 0.06b | 1.09bc | 0.20b |
|  | ADT | 38.7a | 0.04b | 0.88c | 0.20b |
| SY63 | CK | 23.8a | 0.09a | 1.67a | 0.23a |
|  | HNT | 23.3a | 0.09a | 1.57a | 0.20ab |
|  | HDT | 22.5a | 0.05b | 1.00b | 0.18bc |
|  | ADT | 26.6a | 0.04b | 0.86b | 0.17c |

Data are presented as mean ± SD (*n* = 4). Different letters within a column indicate statistical differences among the four temperature treatments for each variety at the *P* < 0.05 level by the least significant difference (LSD) test. ADT, high whole-day temperature treatment; CK, control; CTKs, total cytokinins; HDT, high daytime temperature treatment; HHZ, Huanghuazhan; HNT, high nighttime temperature treatment; LYPJ, Liangyoupeijiu; N22, Nagina22; SY63, Shanyou 63.
